# Titania Nanotubes-Bonded Sulfamic Acid as an Efficient Heterogeneous Catalyst for the Synthesis of *n*-Butyl Levulinate

**DOI:** 10.3389/fchem.2022.894965

**Published:** 2022-05-02

**Authors:** Shuolin Zhou, Min Lei, Junzhuo Bai, Xianxiang Liu, Lu Wu, Min Long, Keying Huang, Dulin Yin

**Affiliations:** ^1^ School of Elementary Education, Changsha Normal University, Changsha, China; ^2^ National and Local Joint Engineering Laboratory for New Petro-chemical Materials and Fine Utilization of Resources, Key Laboratory of the Assembly and Application of Organic Functional Molecules of Hunan Province, College of Chemistry and Chemical Engineering, Hunan Normal University, Changsha, China

**Keywords:** TiO_2_ nanotubes, heterogenous catalyst, levulinate esters, esterification, alcoholysis

## Abstract

The titania nanotubes-bonded sulfamic acid (TNTs-NHSO_3_H) catalyst was designed and successfully fabricated by the post-synthesis modification method. The as-prepared catalyst was characterized by a variety of characterization techniques, including Fourier transform infrared (FT-IR) spectroscopy, X-ray photoelectron spectroscopy (XPS), transmission electron microscopy (TEM), scanning electron microscopy (SEM), X-ray diffraction (XRD) analysis, and thermogravimetry-differential thermal gravimetry (TG-DTG). The crystal structure of the TNTs still maintained during the modification process. Although the BET surface area was decreased, the amount of Brønsted acid sites can be efficiently fabricated on the TNTs. The catalytic activity of TNTs-NHSO_3_H was examined for the synthesis of *n*-butyl levulinate (BL) from levulinic acid (LA) and furfuryl alcohol (FA). A relatively high selectivity (99.6%) at 99.3% LA conversion was achieved for esterification of levulinic acid owing to the strong Brønsted acidity sites. And also, the TNTs-NHSO_3_H catalyst exhibited a higher reactivity for alcoholysis of FA and the yield of BL reached 90.4% with 100% FA conversion was obtained under the mild conditions.

## Introduction

The increasing worldwide fossil fuel consumption and the increasing greenhouse gases emission and other environmental hazards have prompted people to seek other energy sources. The efficient conversion of available and diverse biomass resources into biofuel is an attractive and promising strategy (Gaurav et al., 2017; Alonso et al., 2010). Levulinate esters (LAEs), as a typical representative derived from the bio-based feedstock, have received significant attention. Because they have strong stability, high lubricity, good fluidity, and low combustion oxygen demand, which are similar to fatty acid methyl esters in biodiesel (Christensen et al., 2011). In addition, levulinate esters are one of the most potential platform compounds since their molecular structure contains a carbonyl and ester groups which have high reactivity and can be further applied to produce many important chemicals, such as γ-valerolactone (Zhang et al., 2020) and pyrrolidone (Shen et al., 2019). Furthermore, alkyl levulinates are also the crucial chemicals for other industrially important applications, such as green solvents, plasticizers, and flavoring agents (Wu et al., 2021). Hence, the synthesis of levulinate esters has received abundant attention in recent years from academia and industry, and increasing effort has been devoted to the development of synthetic pathways, the design of novel and efficient catalysts, and optimizing the catalytic process (Démolis et al., 2014).

Up to now, there are several developed potential pathways for the synthesis of LAEs in the presence of various acid catalysts (Tian et al., 2021; Liu et al., 2020). Commercially the acid-catalyzed reactions are largely carried out by different mineral acidic catalysts such as H_2_SO_4_, HCl, and H_3_PO_4_, which often suffer from severe corrosion, product separation and purification, waste, and safety problems. From the practical point of view, readily separated and recovered heterogeneous catalysts, therefore, are more environmentally friendly would be desirable. Among heterogeneous catalysts, TiO_2_ nanotubes (TNTs) materials have attracted extensive attention owing to their chemical stability, large surface area, non-toxicity, and low production cost (Roy et al., 2011). In the past few years, TNTs synthesized by a simple alkaline hydrothermal treatment have been used as a solid acid catalyst for various acid-catalyzed organic transformations (Kitano et al., 2010; Li et al., 2015; de Carvalho et al., 2017). The amount and strength of origin acid sites on the TNTs, however, would be limited in the catalytic applications (Adeleye et al., 2021). It is worth noting that TNTs have a high concentration of hydroxyl groups (–OH) on the surface, which makes them flexible in functionalization with a wide range of organic groups, particular with organosiloxane agents. 3-Aminopropyltrimethoxysilane (APTMS) (Duong et al., 2018), 3-aminopropyltriethoxysilane (APTES) (Pontón et al., 2014), [1-(2-amino-ethyl)-3-aminopropyl]trimethoxy silane (AAPTS) (Wang et al., 2013), octadecyltrimethoxysilane (OTS) (Chao et al., 2013), and octadecyltrichlorosilane (ODS) (Niu and Cai, 2009) have been reported in the literature as chemical modifiers of the TNT surface. Furthermore, hydrothermally synthesized TNTs can be further functionalized by the integration of acidic functional groups to increase the acidity of TNTs in our previous work (Zhou L. et al., 2018; Zhou et al., 2019; Zhou et al., 2022). These studies mean that TNTs as a support material that can be rationally designed and modified as a much higher performance solid acid catalyst, which would bring a wide range of catalytic applications. Moreover, hybrid organic-inorganic materials in the catalytic reaction can provide a synergic and cooperative behavior toward chemo selective and favorable products with high yields (Wight and Davis, 2002).

**SCHEME 1:**
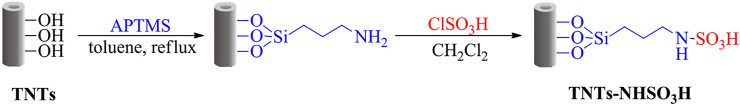
Preparation steps for fabricating titania nanotubes-bonded sulfamic acid.

In pursuit of our recent studies to develop hybrid organic-inorganic solid acid catalysts, the TiO_2_ nanotubes surface was first functionalized with NH_2_ groups through the APTMS grafting and the SO_3_H groups were covalently linked to surface NH_2_ groups by using chlorosulfonic acid as a sulfonating reagent. In the present work, schematic procedures for surface modification on TiO_2_ nanotubes through chemical treatment are presented in Scheme 1. The physicochemical properties of the synthesized TNTs-NHSO_3_H catalyst were characterized by FT-IR, XPS, SEM, TEM, XRD, TGA, and N_2_ physisorption techniques. The potential catalytic activity of TNTs-NHSO_3_H was investigated in the synthesis of high value-added *n*-butyl levulinate from esterification of levulinic acid (LA) and alcoholysis of furfuryl alcohol (FA), respectively.

## Experimental

### Materials

TiO_2_ nanoparticles (anatase, >99.9%), 3-amino-propyltrimethoxysilane (APTMS), LA, and FA were purchased from Aladdin (Shanghai, China) and used as received. Toluene was purchased from Sinopharm Chemical Reagent Co., Ltd. (Shanghai, China) and was freshly distilled before use. Other reagents were of analytical grade and used without further purification. Experimental water was prepared by a Millipore Milli-Q pure water system.

### Catalyst Preparation

#### Preparation of TNTs

TNTs were synthesized by a hydrothermal synthesis method, according to the reported literature with some slight modifications (Kasuga et al., 1999). 2.0 g of TiO_2_ nanoparticles with a particle size of 60 nm were mixed with 60 ml of 10 mol/L NaOH aqueous solution, followed by hydrothermal treatment at 150 C in a Teflon-lined autoclave for 24 h. After cooling, the treated powders were thoroughly washed with 0.1 mol/L HCl and water until the pH value of the washing solution reached 7.0, and subsequently filtered by 0.22 μm filters and dried one night at 80 C under vacuum. The obtained material is labeled as TNTs.

#### Preparation of TNTs-NHSO_3_H

1 g of TNTs was suspended in 20 ml of dry toluene in a 50 ml round-flask and then 3-amino-propyltrimethoxysilane was added. The mixture was refluxed by stirring for 24 h. After the reaction was finished, the solid product was collected by centrifugation, washed three times with chloroform and acetone, respectively, and then dried in a vacuum oven at 80°C. The obtained solid powder was preloaded with 30 ml of dry dichloromethane in a suction flask equipped with a constant-pressure dropping funnel. A certain amount of chlorosulfonic acid was added dropwise over a period of 30 min at room temperature while the mixture was stirred slowly in an ice bath. After the addition was completed, the mixture was further stirred for 4 h. Then, the dichloromethane was removed, and the solid sample was washed with ethanol and dried in a vacuum at 70 C. The resultant solid was designed as TNTs-NHSO_3_H.

### Characterization

Fourier transform infrared spectra (FT-IR) of the samples were collected by the KBr pellet technique on a Nicolet 370 infrared spectrophotometer (Thermo Nicolet, American) in the range 500–4,000 cm^−1^. X-ray photoelectron spectroscopy (XPS) was tested by a Thermo fisher scientific using Mono Al Kα radiation, and binding energies were calibrated at 12.0 kV and 6.0 mA, respectively. The morphologies of the catalyst were investigated by using transmission scanning microscopy (TEM) with an acceleration voltage of 200 kV (JEOL 2100, Japan). The surface morphology of the TNTs-NHSO_3_H catalyst was observed using a scanning electron microscope (SEM, Carl Zeiss Sigma-HD). X-ray diffraction (XRD) studies were carried out using a Bruker diffractometer with Cu Kα radiation to survey the crystal structure over a 2θ range of 10–80°. N_2_ adsorption-desorption isotherms were recorded with an ASAP 2400 physisorption instrument made by Micromeritics Corporation (American). Prior to measurement, all samples were dried under vacuum at 333 K for 24 h. Pore size distribution was calculated using the Barrett–Joyner–Halenda (BJH) method. Thermogravimetric analysis (TGA) curves were recorded in airflow on a Netzsch Model STA 409 PC instrument with a heating rate of 20K/min from room temperature to 973 K using α-Al_2_O_3_ as the standard material. The number of acid sites in each sample was determined by the acid-base titration method.

### Catalytic Test

#### Esterification of LA With *n*-Butanol

In a typical procedure, the mixture of LA (3 mmol) and *n*-butanol (30 mmol) together with the catalyst (5 wt% of LA) were added to a round-bottom flask and was heated at the required reaction temperature in an oil bath under solvent-free condition. At periodic intervals, 0.2 ml of the reaction solution was withdrawn and centrifuged to separate the catalyst and was analyzed by Gas chromatography (GC).

#### Alcoholysis of FA With *n*-Butanol

In a typical run, 1 mmol of FA and 5 ml of *n*-butanol together with a prescribed amount of the catalyst were added to a 25 ml round bottom flask equipped with a reflux condenser under atmospheric pressure. The round bottom flask was heated in an oil bath at reaction temperatures ranging from 90–120°C. At periodic intervals, the reaction mixture was withdrawn and centrifuged to separate the catalyst and was analyzed by Gas chromatography (GC).

### Reaction Intermediates Analysis

The reaction intermediates were identified by GC–MS, a Shimadzu GC system equipped with a capillary (30 m length, 0.32 mm internal diameter, 0.25 μm film thickness). Mass spectrometer conditions were: ionization mode: EI, electron energy 70 Ev; interface temperature: 250°C; ion source temperature: 200°C; mass scan range: 40–640 m/z, solvent delay 3.0 min. The flow rate of the carrier gas (helium) was 1.0 ml min^−1^. A split ratio of 1:50 was used for the injection of 0.2 μl of the solutions. The NIST05 S.LIB library was used for the mass spectrum analysis.

## Results and Discussion

### Characterization of Catalyst

**FIGURE 1:**
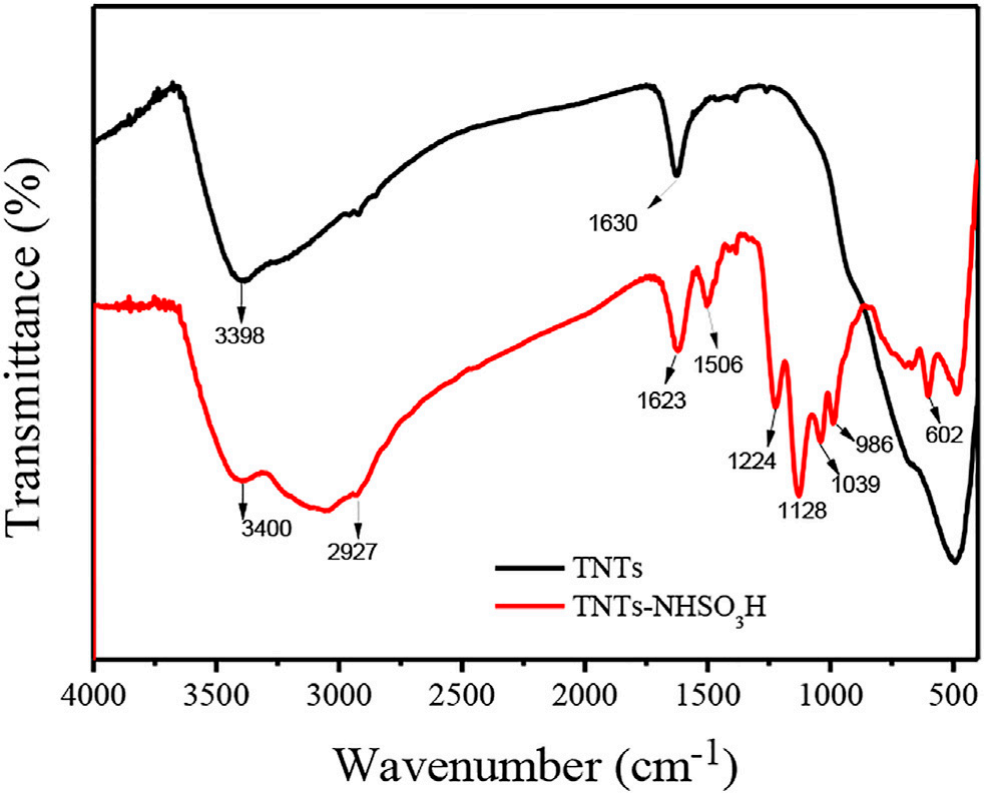
FT-IR spectra of TNTs and TNTs-NHSO_3_H.

FT-IR spectra for TNTs and TNTs-NHSO_3_H samples are presented in Figure 1. Generally, the broad peak present around 3,214–3,600 cm^−1^ and the low-intensity peak at 1,630 cm^−1^ are attributed to the stretching vibration of interlayer water molecules and surface OH groups, respectively. In the case of TNTs, the band below 1,200 cm^−1^ is attributed to Ti-O-Ti vibration and the peak around 490 cm^−1^ can be interpreted as the crystal lattice vibration of TiO_6_ octahedra (Silva et al., 2017). The new absorption peaks in the frequency range of 2,800–2,960 cm^−1^ in the TNTs-NHSO_3_H catalyst are ascribed to the C-H vibration of the aminopropyl segment (Pontón et al., 2014). The peak around 986 cm^−1^ could be interpreted as the Ti-O-Si band. Meanwhile, the band at 1,039 cm^−1^ is ascribed to Si-O stretching vibration. The peaks at 1,128 cm^−1^ and 1,224 cm^−1^ can be assigned to the sulfonyl moiety groups formed by the reaction of chlorosulfonic acid with amino groups (Kassaee et al., 2011).

**FIGURE 2:**
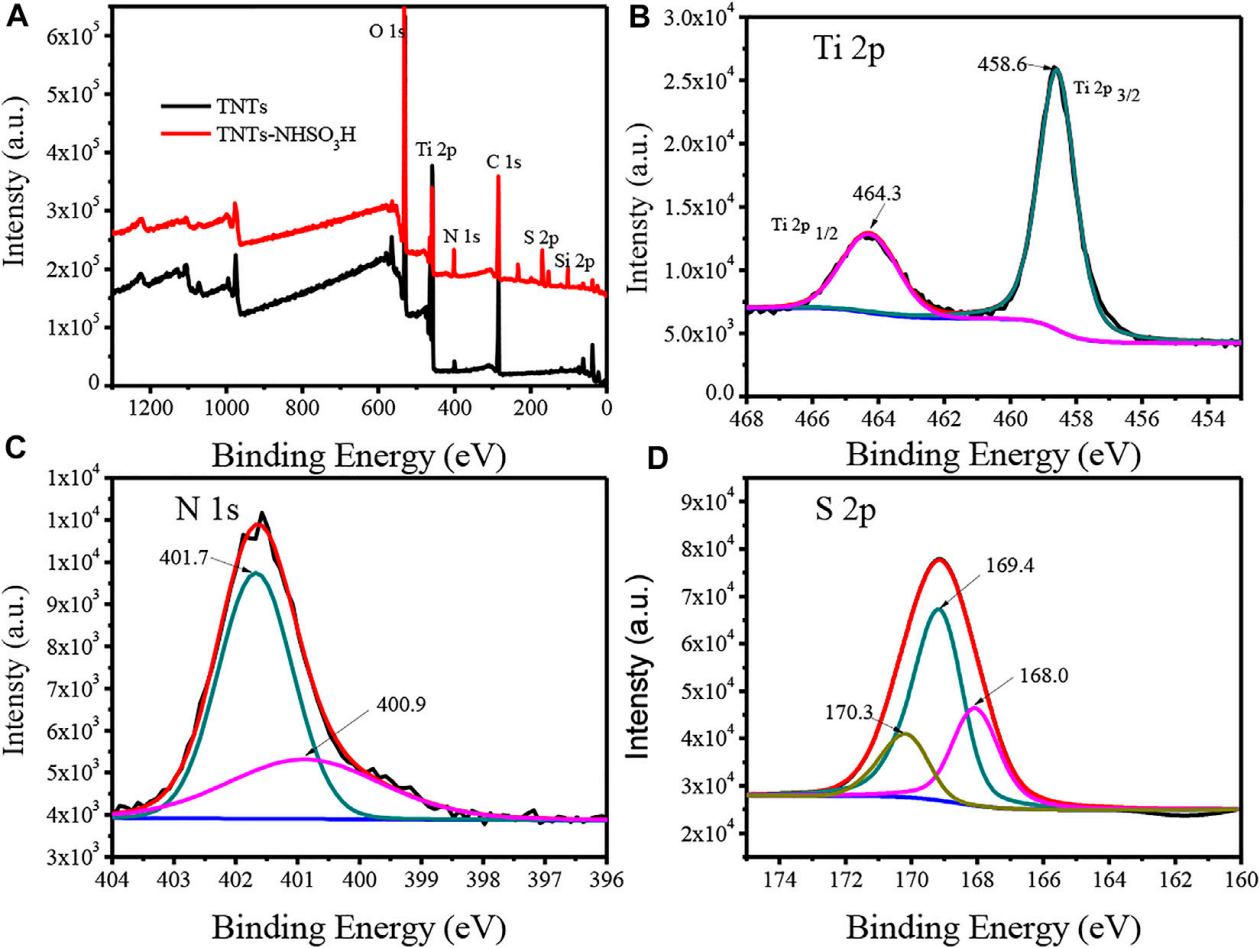
XPS spectra of TNTs and the synthesized TNTs-NHSO_3_H: **(A)** full spectra of samples; **(B)** Ti 2p peaks; **(C)** N 1s peaks; **(D)** S 2p peaks.

The surface chemical compositions and bonding environments for TNTs and TNTs-NHSO_3_H were further analyzed by X-ray photoelectron spectroscopy (XPS), and the results are shown in Figure 2. From the full scan XPS of samples, it can be seen that the TNTs-NHSO_3_H sample is composed of C, N, O, S, Si, and Ti elements (Figure 2A), which are consistent with the elements expected to be obtained by the preparation route. Figure 2B shows the Ti 2p spectra of the TNTs-NHSO_3_H sample. The binding energy of Ti 2p at 458.6 and 464.3 eV is correspond to the Ti 2p3/2 and Ti 2p1/2, respectively. The binding energy of Ti 2p in the catalyst is consistent with that of pure TNTs, supporting that the oxidation state of Ti in the as-prepared TNTs-NHSO_3_H is Ⅳ (Wang et al., 2018). As can be found in Figure 2C, the N 1s signal can be resolved into two peaks. The peak located at 400.9 eV can be assigned to the typical nitrogen present in APTMS. Another peak located at 401.7 eV for the N 1s peak can reasonably be attributed to N-S bonds (Kaid et al., 2020). Figure 2D depicts the XPS spectra of S 2p of TNTs-NHSO_3_H. It is observed that S 2p has a strong peak at 169.4 eV, which is the characteristic peak of typical S=O bonds (Veisi et al., 2016). Two peaks located at 168.0 and 170.3 eV can be assigned to the S-O and S-N, respectively (Song et al., 2015). These results indicate the formation of the desired catalytic system.

The acid amount of the catalyst was determined by the ion-exchanged method using an aqueous solution saturated with NaCl as an exchange agent. The acid density of TNTs-NHSO_3_H is up to 2.27 mmol/g, which implies that a certain amount of Brönsted acidity sites can be constructed on the surface of TNTs.

**FIGURE 3:**
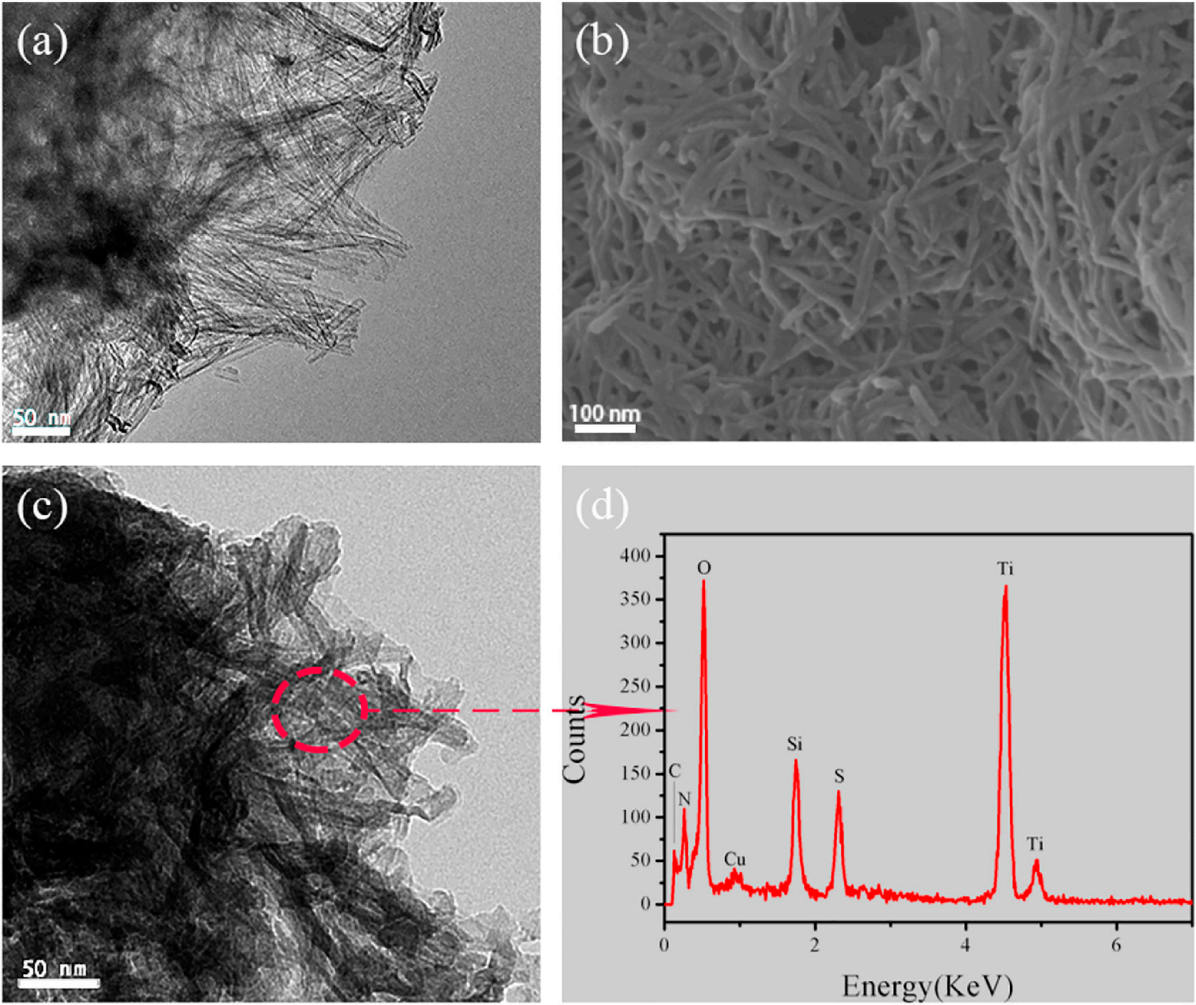
**(A)** Representative TEM image of TNTs. **(B–C)** SEM and TEM images of TNTs-NHSO_3_H. **(D)** EDX spectrum of TNTs-NHSO_3_H.

The morphology of TNTs and TNTs-NHSO_3_H was revealed by SEM and TEM, and the results are displayed in Figure 3. As illustrated by the TEM images, the TNTs sample shows the uniform open-ended tubes can be long up to several hundred nanometers (Figure 3A). It is worthy to note that the morphology of tubes in TNTs-NHSO_3_H was still maintained (Figures 3B,C). The surface of titanate nanotubes was coated with a layer of silicon dioxide in the presence of dry toluene, which is the result of the silanization of surface OH groups with APTMS. This means that more acidity sites can be constructed and immobilized on the TNTs surface by the following functionalization of amino groups in organ siloxane agents. According to the EDX spectrum of an area in Figure 3C, there are five elements, C, N, O, S, Si, and Ti, which can be ascertained in the TNTs-NHSO_3_H (Figure 3D). The percent of N and S is 7.5 and 7 wt%, respectively. This result is consistent with the result previously obtained by XPS, indicating the material was successfully fabricated using a post-synthesis modification strategy.

**FIGURE 4:**
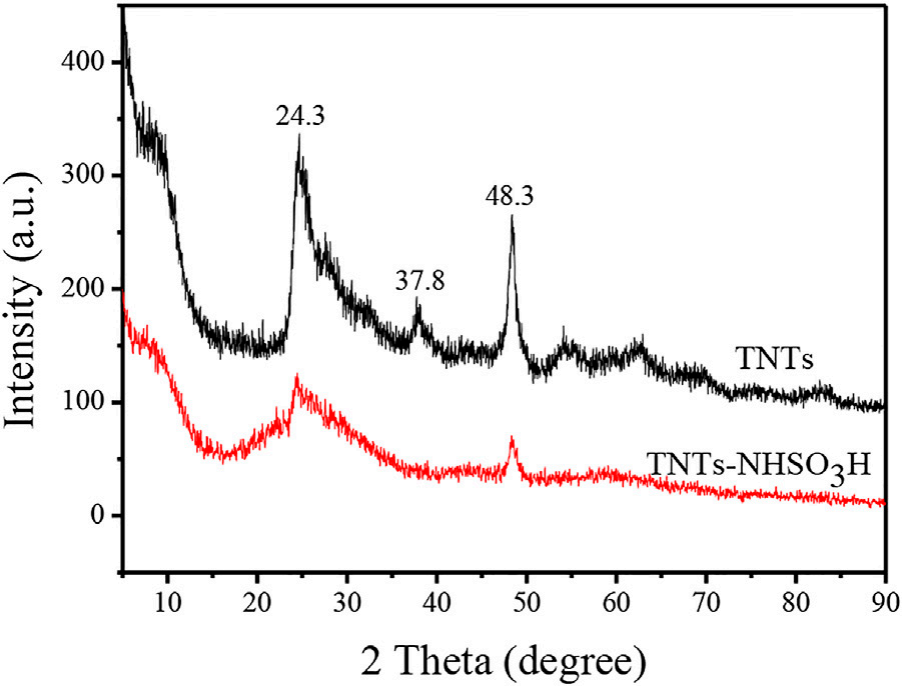
X-ray diffraction patterns of TNTs and TNTs-NHSO_3_H.

The XRD pattern of the TNTs and TNTs-NHSO_3_H are displayed in Figure 4. The TNTs had an XRD pattern that corresponds to the anatase crystal phase of titania. The peak exhibited at a 2θ value of 9.8 indicates the presence of a layered crystal structure formed in the hydrothermal process. It can be seen that all other diffraction peaks at the 2θ value of around 24.3°, 37.8°, and 48.3° are typical characteristic peaks of TNTs (Lu et al., 2017). The XRD pattern of the as-prepared TNTs-NHSO_3_H catalyst can well match the characteristic diffraction peaks of TNTs. It should be noted that there is a slight decrease in the intensity ratios of different peaks, which may be caused by the surface modification during the preparation process.

**FIGURE 5:**
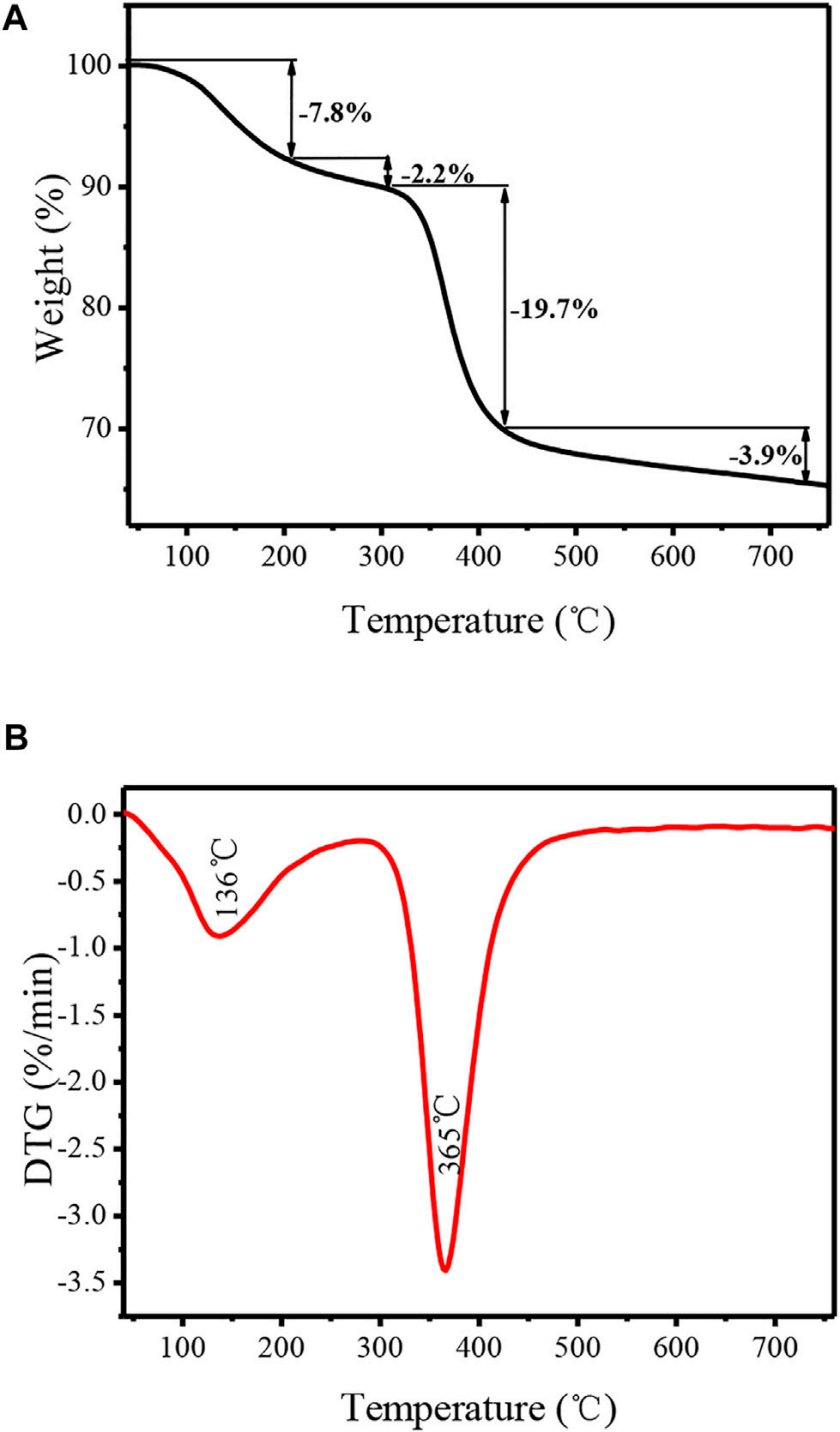
**(A)** TG and **(B)** DTG of analysis of the TNTs-NHSO_3_H catalyst.

In order to further study the surface modification of TiO_2_ nanotubes by organic functional groups, the catalyst was characterized by a thermogravimetric analyzer, and the obtained thermogravimetric curves are presented in Figure 5. It can be seen that a significant weight loss in the range of 100–200 C can be attributed to the elimination of water molecule which was physically adsorbed on the surface and into the pores of TNTs. An endothermic peak with a maximum of136°C can be observed on the corresponding DTG curve. Also, there is a slight weight loss in the range of 200–300 C, which possibly corresponds to the dehydroxylation of TNTs. This phenomenon is consistent with the reported values for the TNT material (Preda et al., 2015). There is an obvious weight loss that started at 300°C, which is mainly attributed to the decomposition of the covalently bound organic functional groups from TNTs-NHSO_3_H. The weight loss of approximately 19.7% in the range of 300–430°C is due to the removal of the organic motif. Furthermore, a strong and sharp endothermic DTG signal was observed at 365°C. In addition, the observed weight loss of approximately 3.9% occurs between 430 and 600°C which may be related to the thermal decomposition of the organic residues. From the TG/DTG analysis, it is understood that the TNTs-NHSO_3_H catalyst has greater thermal stability, which confirms that it can be used for organic reaction temperatures below 300°C.

**FIGURE 6:**
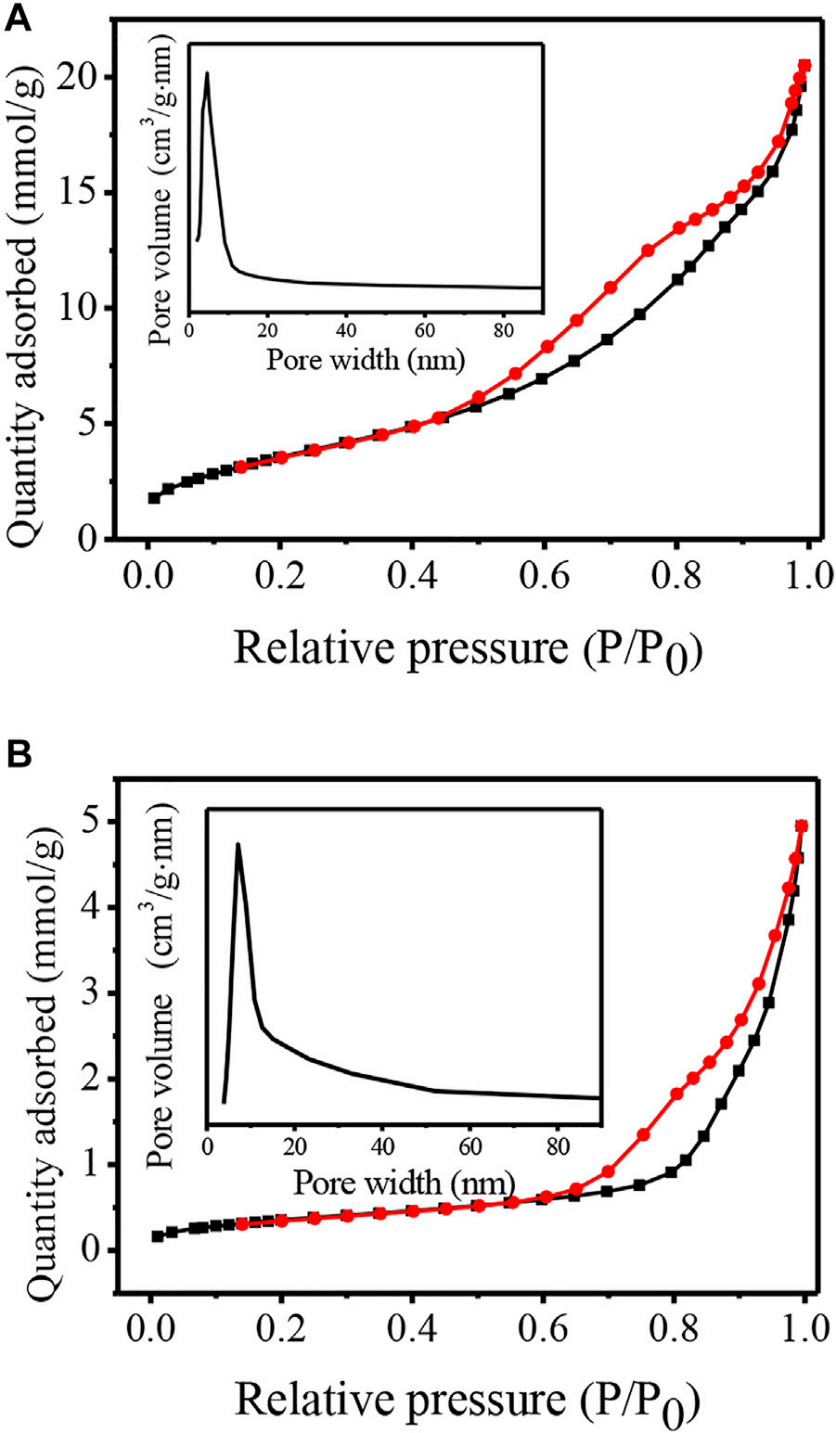
N_2_ adsorption–desorption isotherms and pore size distributions (inset) for **(A)** TNTs and **(B)** TNTs-NHSO_3_H.

Figure 6 shows the N_2_ adsorption-desorption isotherms and the corresponding pore size distribution curves of TNTs and TNTs-NHSO_3_H. According to IUPAC classification, the TNT material exhibits the typical type IV isotherm with a hysteresis loop, confirming the mesoporous nature of TNTs. The surface area of TNTs is 300 m^2^/g. As shown in Figure 6A(inset), the pore size distribution presents the maximum positioned at 4.6 nm, corresponding to the inner nanotubular cavity of TNTs. This result suggests that TNTs had a uniform nanotubular structure. After functionalization of the parent material, the remarkable change in the N_2_ adsorption-desorption isotherm curve was observed in Figure 6B. It is worth noting that the surface area of TNTs-NHSO_3_H decreased to 29 m^2^/g. It is inferred that a part of the organosiloxane agents was confined inside the nanotubes, especially near the ends of the nanotubes, as TNTs possess a large fraction of voids in the interior, which resulted in the partial blocking of the tubular channel of TNTs-NHSO_3_H. Although the BET surface area of TNTs-NHSO_3_H is decreased in comparison to that of pure TNTs, it is still favorable for the acid-catalyzed reactions due to the more accessible acidic sites on the inside or outside of TNTs.

### Catalytic Performances

#### Esterification of LA With *n*-Butanol

**SCHEME 2:**
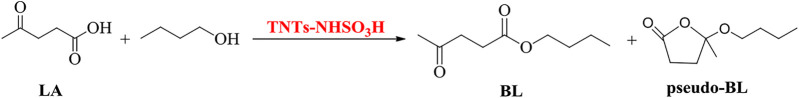
Esterification of LA with *n*-butanol over the TNTs-NHSO_3_H catalyst.

**FIGURE 7:**
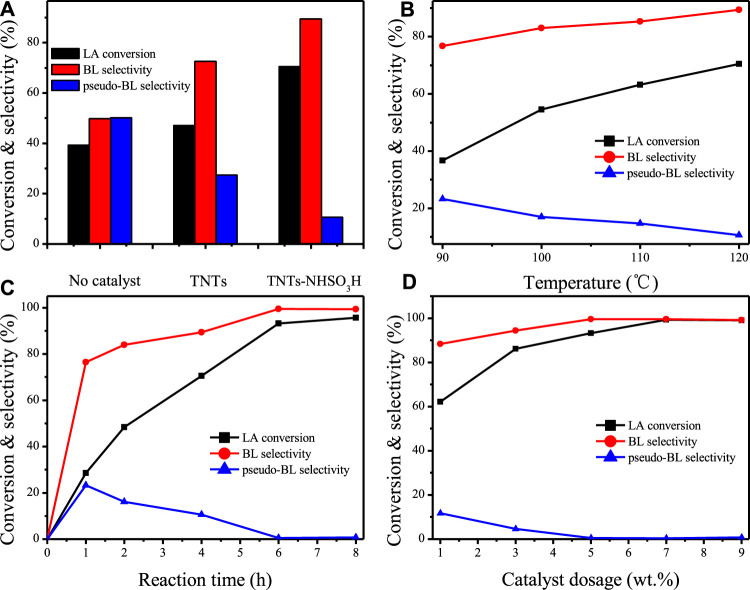
**(A)** Catalytic activities over TNTs and TNTs-NHSO_3_H. Reaction conditions: 5 wt% catalyst dosage, reaction time 4h, 120°C. **(B)** Effect of reaction temperature. Reaction conditions: 5 wt% catalyst dosage, reaction time 4 h. **(C)** Effect of reaction time. Reaction conditions: 5 wt% catalyst dosage, 120°C. **(D)** Effect of catalyst dosage. Reaction conditions: 120°C, reaction time 6 h.

The catalytic performance of TNTs-NHSO_3_H was first tested for the esterification of LA with *n*-butanol (Scheme 2) and the results are illustrated in Figure 7. The reaction products, *n*-butyl levulinate (BL) and *p*seudo-n-butyl levulinate (*pseudo*-BL) were detected by GC-MS techniques. Similar results were also observed by several authors (Ciptonugroho et al., 2016; Chaffey et al., 2021). As shown in Figure 7A remarkable catalytic performance of TNTs-NHSO_3_H was observed since LA conversion (70.5%) with high selectivity (89.4%) to BL was achieved. The appearance of Brønsted acid centers in the TNTs-NHSO_3_H catalyst is the primary reason for higher catalytic performance. To appraise the effects of reaction temperature, systematic studies were carried out at different temperature ranges from 90 to 120°C for 4 h (Figure 7B). The relative increase in both the LA conversion and BL selectivity was obtained when the temperature was raised from 90 to 120°C. This result suggests that the relatively high reaction temperature is favorable for the conversion of *pseudo*-BL to BL and thereby improving the selectivity of BL. It should be noted that TNTs-NHSO_3_H showed good catalytic activity at 120°C giving high conversion and high selectivity. Figure 7C shows the reaction time on LA conversion and BL selectivity in the range of 1–8 h at 120°C. It is found that the LA conversion and BL selectivity was increased with reaction time. A relatively high selectivity (99.5%) at 93.2% LA conversion was achieved after 6 h. However, there was no significant increase in conversion or yield with further prolonged time to 8 h. The effect of catalyst dosage on the conversion and yield was estimated while keeping the other variables constant and the results are plotted in Figure 7D. It was found that the LA conversion and the BL yield increased with the increasing amount of the catalyst. The optimum catalyst weight was found to be 7 wt% with 99.3% LA conversion because the further increase of the amount of catalyst exhibited a slight increment in conversion as well as yield.

#### Alcoholysis of FA With *n*-Butanol

**SCHEME 3:**
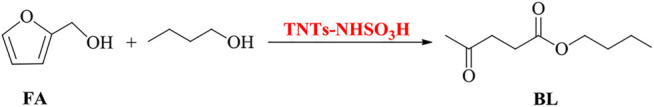
Alcoholysis of FA with n-butanol over the TNTs-NHSO_3_H catalyst.

**FIGURE 8:**
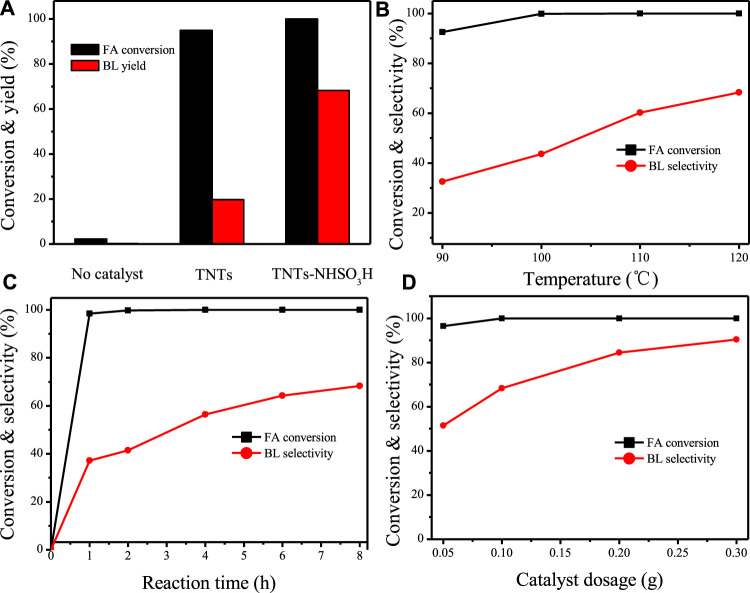
**(A)** Catalytic activities over TNTs and TNTs-NHSO_3_H. Reaction conditions: 0.1 g catalyst, reaction time 8h, 120°C. **(B)** Effect of reaction temperature. Reaction conditions: 0.1 g catalyst, reaction time 8 h. **(C)** Effect of reaction time. Reaction conditions: 0.1 catalysts, 120°C. **(D)** Effect of catalyst dosage. Reaction conditions: 120°C, reaction time 8 h.

The catalytic performance of the TNTs-NHSO_3_H catalyst was further tested by synthesis of *n*-butyl levulinate from FA (Scheme 3) and the results are illustrated in Figure 8. A 19.7% yield of BL was achieved in the presence of the TNT sample. The catalytic activity of the TNTs was attributed to the number of Lewis acidic sites on its surface (Kitano et al., 2010). It is important to mention that the catalyst TNTs-NHSO_3_H exhibited high activity for alcoholysis of FA with *n*-butanol giving a high yield of BL (Figure 8A). Based on this result, the effects of the variable parameters, including reaction temperature, reaction time, and catalyst dosage on the alcoholysis of FA to BL were investigated. As illustrated in Figure 8B, the BL yield was strongly affected by the reaction temperature. At a low reaction temperature (90°C), the BL yield was relatively low (32.5%). However, when the reaction temperature was increased from 90 to 120 C, the yield of BL increased significantly. This is because the higher reaction temperature facilitates the conversion of FA into BL. However, when the reaction temperature was further increased, there was no significant increase in the BL yield. This may be attributed to the undesired side reactions that occurred at higher temperatures (Huang et al., 2016). Figure 8C displayed the effect of reaction time on the production of BL. Complete conversion was reached around 1 h, whereas the initial yield of BL was low. It is noteworthy that the yield of BL gradually increases with an increase in reaction time from 1–8 h. There is no remarkable change after 6 h, and the maximum yield (68.3%) was obtained at 8 h. As shown in Figure 8D, BL yield was highly affected by catalyst dosage. It can be easily found that by increasing the catalyst amount up to 0.3 g, the yield of BL reached 90.4% with 100% FA conversion. This clearly indicates the fact that the increase of the loading of catalyst provides the more available acidic sites, thereby promoting the conversion of intermediate product to the target product.

Additionally, the catalytic performance of TNTs-NHSO_3_H was better than that of other systems listed in Table 1. It is clear from the table that TNTs-NHSO_3_H worked as an acid catalyst exhibiting excellent catalytic performance in the synthesis of n-butyl levulinate from both LA and FA. More works to tune the catalytic properties of the catalyst and to characterize the surface acid properties are underway.

**TABLE 1:** Comparison of TNTs-NHSO_3_H with the reported catalyst.

Catalyst	Substrate	Temp. (°C)	Time (h)	BL yield (%)	Ref
TNTs-NHSO_3_H	LA	120	6	98.9	This work
Meso-ZSM-5	LA	120	5	82.2	Morawala et al. (2019)
H_3_PW_12_O_40_/Al-MCM-41	LA	120	4	90.0	Najafi Chermahini and Nazeri, (2017)
Sulfonated glucose-derived amorphous carbon	LA	100	4	90.5	Yang et al. (2018)
SnTUD_-1_	LA	120	4	90.5	Pachamuthu et al. (2019)
NER@3DOM/m-OS	LA	40	12	74.6	Zhou S et al. (2018b)
TNTs-NHSO_3_H	FA	120	8	90.4	This work
SMWP	FA	120	5	90.6	Yang et al. (2020)
[MIMBS]_5_ [AlW_12_O_40_ ]	FA	120	6	94	Hao et al. (2017)
UiO-66(Hf)-SO_3_H	FA	120	4	72	Gupta and Kantam, (2019)
KCC-1/Pr-SO_3_H	FA	120	3	81.9	Mohammadbagheri and Najafi Chermahini, (2018)
GC-PTSA-AC	FA	120	4	91.0	Yang et al. (2019)

#### Reusability

**FIGURE 9:**
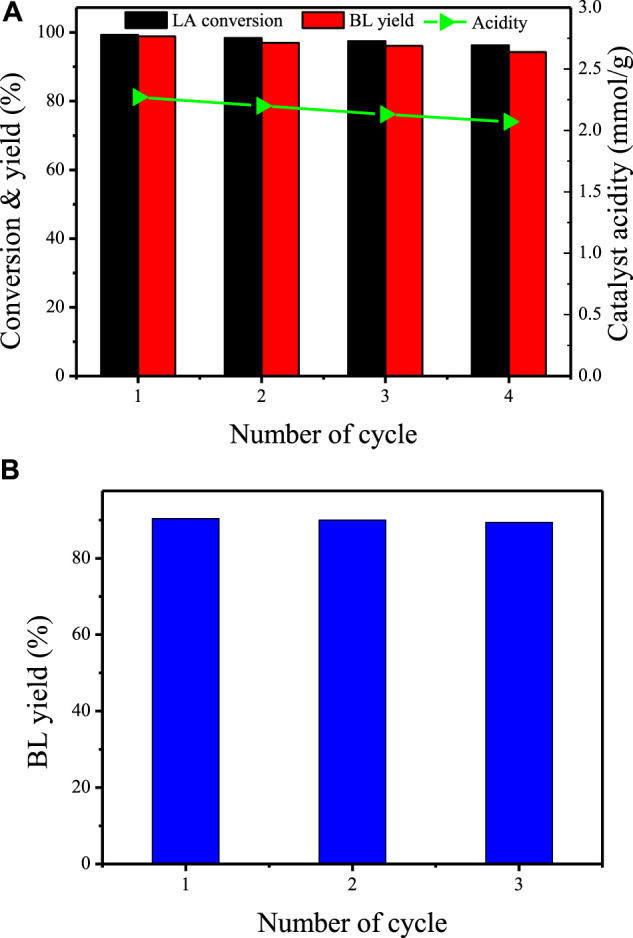
Reusability of TNTs-NHSO_3_H toward **(A)** esterification of LA and **(B)** alcoholysis of FA. Reaction conditions for **(A)**: LA 3 mmol; n-butanol 30 mmol; 120°C; 6h; 7wt% catalyst. Reaction conditions for **(B)**: FA 1 mmol; n-butanol 5 ml; 120°C; 8h; 0.3 g catalyst.

To understand the recyclability of TNTs-NHSO_3_H, the consecutive runs under the optimum conditions were performed in the esterification of levulinic acid with n-butanol. After each cycle, the spent catalyst was recovered by centrifugation, washed with ethanol, and dried for the next run. As presented in Figure 9A, the activity loss of TNTs-NHSO_3_H is neglectable after four cycles, and a yield of the desired product BL was still maintained at 95.6%. It is worthy to note that the acidity of the catalyst declined from 1.28 to 1.06 mmol g^−1^ after four consecutive recycles. The FT-IR analysis of the spent catalyst (Supplementary Figure S1) also confirmed that the active functional groups of the catalyst were relatively stable, providing the evidence for heterogeneous nature of TNTs-NHSO_3_H. In addition, repeated runs of the alcoholysis reaction of FA with n-butanol were carried out under the above optimum conditions, and the results are displayed in Figure 9B. It was found that the catalyst was reused with a slight decrease in BL yield. This may be due to the black carbonaceous species, formed by the polymerization of the FA (Zhu et al., 2017), decreasing the accessibility of the substrates to the active sites.

## Conclusion

In this study, titanate nanotubes were obtained through hydrothermal synthesis and then covalently linked sulfamic groups through the APTMS grafting and chlorosulfonic acid as a sulfonating reagent. The catalyst was fully characterized by various physicochemical characterization techniques, and the corresponding results suggest that the strong sulfonic acid groups were successfully grafted on the titanate nanotube surface. The as-prepared TNTs-NHSO_3_H catalyst was used as a Brønsted solid acid for the synthesis of *n*-butyl levulinate from LA and FA. The excellent acid catalytic performance was observed in the synthesis of *n*-butyl levulinate from both the esterification of LA and the alcoholysis of FA under mild conditions. These results demonstrated that the obtained catalyst can be utilized as a potential candidate for the synthesis of LAEs and other value-added chemicals from biomass.

## Data Availability

The original contributions presented in the study are included in the article/Supplementary Material, further inquiries can be directed to the corresponding author.
