# Supplementary material for: Titania Nanotubes-Bonded Sulfamic Acid as an Efficient Heterogeneous Catalyst for the Synthesis of n-Butyl Levulinate
Source: Front Chem. 2022 May 2;10:894965. doi: 10.3389/fchem.2022.894965 (PMC9108267; doi:10.3389/fchem.2022.894965)
Supplement: Supplementary file 1 [file DataSheet1.DOCX]

Supplementary Material

**Supplementary Figure 1.** FT-IR spectra of the used TNTs-NHSO_3_H
